# High Expression of Endogenous Retroviral *Envelope* Gene in the Equine Fetal Part of the Placenta

**DOI:** 10.1371/journal.pone.0155603

**Published:** 2016-05-13

**Authors:** Valentina Stefanetti, Maria Luisa Marenzoni, Fabrizio Passamonti, Katia Cappelli, Koldo Garcia-Etxebarria, Mauro Coletti, Stefano Capomaccio

**Affiliations:** 1 Department of Veterinary Medicine, University of Perugia, Perugia, Italy; 2 Department of Genetics, Physical Anthropology and Animal Physiology, University of Basque Country-UPV/EHU, BioCruces Health Research Institute, Leioa, Spain; The University of Melbourne, AUSTRALIA

## Abstract

Endogenous retroviruses (ERVs) are proviral phases of exogenous retroviruses that have co-evolved with vertebrate genomes for millions of years. Previous studies have identified the envelope (*env*) protein genes of retroviral origin preferentially expressed in the placenta which suggests a role in placentation based on their membrane fusogenic capacity and therefore they have been named *syncytins*. Until now, all the characterized *syncytins* have been associated with three invasive placentation types: the endotheliochorial (Carnivora), the synepitheliochorial (Ruminantia), and the hemochorial placentation (human, mouse) where they play a role in the syncytiotrophoblast formation. The purpose of the present study was to evaluate whether EqERV *env* RNA is expressed in horse tissues as well and investigate if the horse, possessing an epitheliochorial placenta, has “captured” a common retroviral *env* gene with syncytin-like properties in placental tissues. Interestingly, although in the equine placenta there is no syncytiotrophoblast layer at the maternal-fetal interface, our results showed that EqERV *env* RNA is highly expressed at that level, as expected for a candidate syncytin-like gene but with reduced abundance in the other somatic tissues (nearly 30-fold lower) thus suggesting a possible role in the placental tissue. Although the horse is one of the few domestic animals with a sequenced genome, few studies have been conducted about the EqERV and their expression in placental tissue has never been investigated.

## Introduction

Endogenous retroviruses (ERVs) are genomic elements present in a wide range of hosts from basal vertebrates to mammals [1]. During the course of evolution, exogenous retroviruses have copied themselves into the germ line resulting in integrated endogenous retroviruses that are transmitted vertically to the offspring as Mendelian genes [1] and make up approximately 8–10% of the host genome in mammals [2].

According to the phylogenetic relationships with exogenous retroviruses, ERVs are divided into three classes. Class I ERVs are related to the *Gammaretrovirus* and *Epsilonretrovirus* genera; Class II ERVs are related to the *Alpharetrovirus*, *Betaretrovirus*, *Deltaretrovirus*, and *Lentivirus* genera; and Class III ERVs have a distant relationship with the *Spumaretrovirus* genus [3,4]. A complete ERV provirus presents the same general “three genes structure” as an exogenous retrovirus: *gag*, *pol* and *env* [5]. These genes are flanked by two non-coding long terminal repeats (LTRs) which are control regions containing promoters, enhancers and polyadenylation signals [1].

Most of the ERVs have been altered by genetic mutations, insertions and/or deletions, and recombinations, either passively or in response to host-defence mechanisms. Consequently, the majority of endogenous retroviral sequences are inactivated [1,6]. However, several ERVs maintain at least some intact open reading frames (ORF) with viral protein expression associated with either beneficial or detrimental effects for the host [7]. Conservation of ERV genes through evolution suggests, however, that they have provided beneficial effects to their hosts’ survival. As described in detail previously [2], a striking example of such positive selection is demonstrated by the *syncytin* genes that have been acquired by eutherian mammals for a key role in placenta formation.

In humans, specific *env* genes contained within an endogenous retrovirus insertion have retained an intact ORF and are preferentially expressed in the multinucleate syncytiotrophoblast layer of the placenta [8]. These two genes, named “*syncytin-1* and *-2*”, are capable of promoting trophoblast cell fusion in vitro and have been functionally conserved for more than 25 and 40 million years of evolution respectively [8,9]. One of them, *syncitin-2*, also displays immunosuppressive activity [10]. Interestingly, similar genes have also been reported to be expressed, preferably in the placenta, in various eutherian mammals including Carnivores (*syncytin-Car1*) [11], Rodents (*syncytin-A* and *–B*) [12], Lagomorphs (*syncytin-Ory1*) [13] and Ruminants (*syncytin-Rum1*) [6]. All identified *syncytins*, although having a different origin and integrating at different genomic locations, share closely related functional properties suggesting a convergent evolutionary process [14]. There are several examples of the wide impact of ERVs in host’s biology: it has been recently demonstrated that the mouse *syncytin-A* and–*B* genes are essential for trophoblast cell differentiation and syncytiotrophoblast morphogenesis during placental development [15] and that an endogenous sheep retrovirus (enJSRVs) has a biological role in preimplantation ovine conceptus development and placentation [16].

One challenging hypothesis is that this stochastic capture of exogenous-origin genes might play a role in establishing the functional and structural diversity of mammalian placentation. Indeed, as described in detail previously [2], one of the major divergent aspects among modern eutherian mammal placentae is the extent of invasion of the maternal uterine tissues by the trophoblast covering the blastocyst at the time of implantation which leads to four major placental types. In the epitheliochorial placentation (horse and pig), the fetal trophoblast cells are simply apposed to the intact maternal epithelium. Until now, all the characterized syncytins have been associated with the endotheliochorial (carnivores), synepitheliochorial (ruminants) and hemochorial placentation (humans, rodents, lagomorphs). These are three highly invasive placentation types where the syncytins are involved in syncytiotrophoblast formation.

Although the horse is one of the few domestic animals whose genome has been sequenced [17], data for specific repetitive sequences are scarce. A full-length beta retrovirus was detected on the equine chromosome 5. This provirus, named EqERV-beta1, is the first endogenous equine retrovirus discovered in the genome of the horse [18]. Through *in silico* analysis, Garcia-Etxebarria and colleagues detected and characterized ERVs in the horse genome. They used a comparison of three different bioinformatics methods which showed that ERVs detection and distribution depends on the discovery algorithm used [4]. However, only a few studies have been made about equine ERVs (EqERVs) and their expression in placental tissue has never been investigated.

The aim of the present study was to appraise the EqERVs *env* expression in different equine tissues and to evaluate whether the horse, possessing an epitheliochorial placenta where the fetal and maternal tissues are simply apposed, has “captured” a common retroviral *env* gene with syncytin-like properties.

## Materials and Methods

### Selection of full-length ERVs

In order to investigate the expression of EqERVs in target tissues, a pre-selection of these elements was conducted through an *in silico* analysis. Exploiting previous knowledge obtained from Garcia-Etxebarria et al. [4], which sums up the results of three discovery algorithms (LTR_STRUCT, BLAST and Retrotector), we reduced the dataset by selecting only elements predicted by at least two different methods. Further reduction was achieved by choosing retroviral *env* sequences homologous to surface (SU) *env* of known EqERVs thus ending with a smaller dataset of full-length candidates. ORF longer than 300 amino acids (from start to stop codon) were searched for with NCBI ORF finder (http://www.ncbi.nlm.nih.gov/gorf/gorf.html). Downstream analyses were conducted on positive entries.

### Primer design

To avoid amplification of unwanted retroviral elements, a multiple alignment with the 15 full-length candidates was performed to pinpoint maximum diversity regions in which to safely design the PCR primers. Primers were designed on the *env* region of the candidate full-length ERV using Primer3 software [19] and the sequences are:

- EqERV_chr5_FOR 5’-ACTGGACGTCTTGACAGCAG-3’
- EqERV_chr5_REV 5’-TAGGGGAGGGTCAGACATGG-3’

A BLAT analysis was performed to verify the amplicon uniqueness and primers locus specificity.

### Study design and sample collection

In this study, different horse tissues were sampled to investigate the EqERV *env* expression using two groups of animals. In the first group, composed of 15 horses (mean age = 4.7 years; median = 1; range = 1–28, 9 females and 6 males), non-placental organs (lung, liver, kidney and spleen), were obtained *post-mortem*. In the second group, fetal parts of the placentae of 15 different mares, (mean age = 11.3; median = 11; range = 6–19) were collected immediately after an eutocic delivery at the end of a physiological gestation.

Three parts of each organ were randomly collected and immediately frozen in liquid nitrogen before being stored at -80°C until used for RNA extraction.

### RNA extraction and qRT-PCR

For each sample, total RNA was extracted from 100 mg of ground tissue using the Trizol Plus RNA purification kit (Ambion, Life Technologies), according to the manufacturer’s instructions. RNA concentration was assessed using the NanoDrop^®^ spectrophotometer and the integrity of RNA was examined by electrophoresis in a denaturing 1% agarose gel.

One microgram of total RNA was treated with DNase I (Invitrogen) and successful DNA removal was verified by qPCR on 2μl of treated RNA with the same EqERVs primer pair previously described and using the following thermal cycle: initial denaturation at 98°C for 3 min, followed by 35 cycles of 98°C for 5 s and 61°C for 1 min.

Purified RNA (350 ng) was reverse transcribed into cDNA using a mixed priming strategy (Oligo dT + Random Hexamers) with PrimeScript RT Reagent Kit (Takara) and incubated under the following conditions: 37°C for 15 min and 85°C for 5 s.

### qPCR assay

The qPCR reaction was carried out with 5 μl of a ten-fold diluted cDNA in a final volume of 20 μl using 10 μl of SsoFast^™^ EvaGreen^®^ Supermix, (BioRad). The amplification was performed in a CFX96 Touch instrument (BioRad, Hercules, CA), using the following thermal conditions: 98°C for 3 min, then 40 cycles of 98°C for 5 s and 61°C for 1 min. Amplification uniqueness was confirmed by sequencing.

The expression ratio of the gene of interest was normalized relative to the abundance of two reference genes (*β-actin* and *GAPDH*) [19–21] to adjust for unbalanced samples and corrected for coexpression in the qRT-PCR. Each reaction was run in triplicate and no-template controls (NTC) were included in each run. Normalization was performed using the ΔΔ Cq method [22] that uses geometric mean of Cq values. Technical replicates were averaged and only those samples with standard error lower than 0.2 Cq were maintained. Data analysis was carried out with Bio-Rad CFX Manager software (ver. 3.2.2) and GenEx (ver.6). All values are presented as means with a confidence interval of 99%.

### Statistical analysis

Considering the limited number of animals, the presence of paired and unpaired data and the distribution of the EqERV *env* RNA expression in the tissues, appropriate statistical tests were used.

Differences in the variance of EqERV *env* RNA expression among all the tissues was assessed with the Levene's test.

Pairwise comparison for EqERV *env* RNA expression in paired data (lung, kidney, liver and spleen) were investigated using paired non-parametric tests (Wilcoxon Signed Ranks test and the Friedman test).

Unpaired non-parametric tests (Kruskal Wallis or Mann-Whitney) were used to compare expression data among lung, kidney, liver, spleen and fetal parts of the placenta. The same tests were also used to assess if age or sex had an effect on EqERV *env* expression. Age was further analysed by trying to group the animals into different age categories with testing at two different cut-offs: ≤ 6 or ≤ 10 years. A *P value* < 0.05 was considered significant for the analysis. Data were analysed with R, version 2.8.1 (R, Development Core Team 2007).

### Ethics statement

Part of the samples of the present study (liver, spleen, kidney and lung) were obtained *post-mortem*. All animals were in good general health and were presented for slaughter for reasons unrelated to this study. The horses were slaughtered in February 2015 in an official abattoir (Macello Pubblico Centro Carni, Roma, Italy, IT-CE-1252 M) for non-research purposes, in accordance with EU legislations EC 852/2004, 853/2004 and 854/2004.

The fetal membranes samples were collected by qualified veterinarians immediately after an eutocic delivery with a full placental expulsion at the end of a physiological gestation specifically for the purpose of this study. All sampling was performed as part of post-delivery routine clinical examinations by licenced veterinarians of horses on private farms with written consent from the owners. All sampling procedures complied with national and European regulations and, due to the non-invasive nor experimental-induced sampling, the present study is not subject to approval in the usual way by the Ethical Committee of our Institution.

## Results

From 1948 ERVs loci only 340 have been detected by at least two algorithms used by Garcia-Etxebarria and colleagues [4]. Then only those sequences homologous to surface (SU) *env* of known EqERVs were chosen because previous studies had demonstrated that syncytin was encoded by an *env* gene ending with a smaller dataset of 15 full-length candidates (S1 Table). Among the selected full-length EqERVs, we found only one candidate showing an ORF longer than 300 aa suitable for downstream investigation. This element is located on chromosome 5 (coordinates on equcab2: chr5:27325635–27334495) and it is EqERV4, a member of class I family: namely, an ERV related to *Gammaretroviruses*.

**Fig 1:**
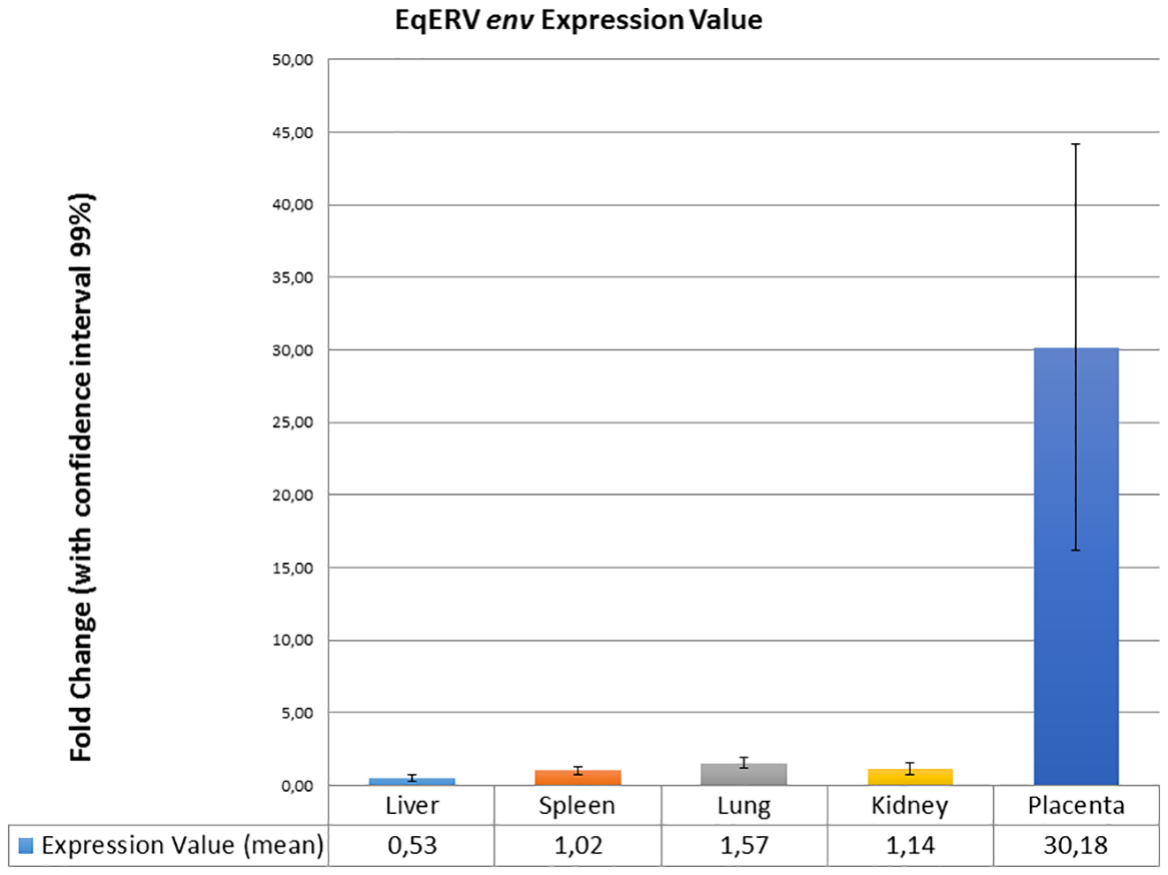
Expression of EqERVs *env* RNAs in normal equine tissues. Each value was normalized to the amount of two reference genes (*β-actin* and *GAPDH*) and was exhibited as means of all the triplicate experiments with confidence interval (CI) 99%. Normalization was performed using the ΔΔ Cq method [22] that uses geometric mean of Cq values. Technical replicates were averaged and only those samples with a standard error lower than 0.2 Cq were maintained.

Due to the high representation of *env*-like sequences in the horse genome, genomic DNA removal from RNA samples was a crucial step for ERV RNA assessment: all the samples showed no amplification curves after 35 qPCR cycles thus confirming successful DNAse treatment. Afterwards, *env* RNA expression level in the fetal part of the equine placenta and in a panel of other tissues was examined. As shown in Fig 1, this gene is highly expressed in the fetal part of the placenta as expected for a candidate syncytin-like gene with reduced abundance in the other somatic tissues (nearly 30-fold lower). Levene’s test showed that the variance of the fetal part of the placental tissues differs significantly from those of liver (*P* = 0.0294), spleen (*P* = 0.0308), lung (*P* = 0.0320), and kidney (*P* = 0.0331) (S2 Table). Interestingly, statistical differences were found in the EqERVs expression between spleen and liver (*P* = 0.008), lung and liver (*P* = 0.001), kidney and liver (*P* = 0.002), and lung and spleen (*P* = 0.004) whereas no difference was found in EqERVs expression between kidney and lung (*P* = 0.053) and kidney and spleen (*P* = 0.3). In the present study, no association was found between the EqERVs expression in the different tissues and the age or the gender of the horses.

To allow comparison with previous works, expression levels were converted to percentage of maximum. We found a 5% of expression in lung, 3.7% in kidney, 3.4% in spleen compared with the fetal part of placenta (S1 Fig).

## Discussion

As complete genome sequences have become available, genome-wide studies of ERVs have been conducted using different bioinformatics approaches in various animal species. Some authors showed that ERVs detection and distribution in the horse genome may depend on what bioinformatic approach is applied [4]. Actually, since each tool gathers non-completely overlapping results, we decided to be conservative and select only the ERVs detected by more than one methodology. Finally, after further evaluations, only one of the selected full-length candidates was analysed as being the result of a relatively recent integration and preserving almost complete coding capacity [23]. As in carnivores, murids and ruminants [6,11,12], the analysed EqERV is a Class I family (*Gammaretrovirus*) although it is not annotated in the horse genome. In ruminants, ERVs from Class II (*Betaretroviruses*) were also described [24].

The qRT-PCR assay revealed that the investigated EqERV *env* RNA has an expression level higher in the placenta (30-fold-change) than in other equine tissues. Interestingly, although the EqERVs *env* expression in non-placental tissues is relatively low, some statistically significant differences have been observed in the analysed tissues: excluding the fetal part of the placenta, the lowest expression was detected in the liver and the highest in the lung. The expression of ERVs has been analysed in domestic animals in several organs by different methods such as qRT-PCR and RNA-Seq. Some studies on ERVs expression have been carried out in pigs, especially in organs relevant for xenotransplantation. Interestingly, similar to our findings, some authors detected among non-placental swine tissues a high expression of the spliced *env* mRNA in the lung and in the spleen and lower expression in the liver [25].

For cats and dogs, *env* genes showed variable expression patterns in the analysis of non-placental tissues [11]. In horses, Brown et al. analysed five tissue samples using RNA transcriptome sequencing (kidney, jejunum, liver, spleen and mesenteric lymph node) which showed that none of the analysed ERVs was expressed [18]. However, the differing results from our study could be linked to the different approaches used and, additionally, reproductive tissues were not analysed in the study of Brown and colleagues. The functional consequences of this different viral gene expression pattern remain unknown thus further studies are required to fully understand the regulatory mechanisms by which EqERVs sequences are more highly expressed in some non-placental tissues than other.

The most interesting result of the present research is the high EqERV *env* expression in the fetal part of the placenta compared with other tissues. Ideally, the same set of tissues would be collected from each animal; however, it was impossible to sample organs (liver, spleen, kidney and lung) and placentas from the same mares because there is an European Union Law (CE 1/2005) that prohibits the transportation of animals during the last period of pregnancy and during the week following the delivery.

Although the exact mechanism by which EqERV *env* expression was suppressed in some non-placental tissues is not known, there could be some epigenetic regulatory systems like those of other ERVs. In humans, in fact, it has been demonstrated that CpG methylation of *syncytin-1* LTR efficiently blocks its transcription in non-placental tissues [26].

With expression values converted to percentage of maximum, direct comparison with previous works in other species is allowed. We noticed that EqERVs *env* in lung, spleen and kidney appear to be expressed at higher levels compared to Ruminants and Carnivora in the same tissue [6,11]. We speculate that these differences could be due to a lower expression of the EqERVs *env* in the fetal part of the placenta rather than higher levels in other tissues, where this transcript is unlikely to exert a function. This can be explained with a lower expression in the horse, where syncytiotrophoblast is absent, or with a bias in the anatomical site of the sample collection. In fact, results of the present study could be affected by the sampling process: different portions randomly collected from the equine fetal part of the placentae were pooled, thus likely decreasing EqERVs *env* expression level with a “dilution” effect. Moreover, as underlined by the variance analysis, the wide “heterogeneity” in the specimens could also explain the lower placenta-expression level when compared with those other animal species sampled in specific placental sites (i.e. the placentomes in Ruminants). This could be verified using a different sampling approach in equine placenta for future research. Previous studies have confirmed that ERV envelope proteins were found in “invasive” placental types and promote the cell-to-cell fusion of trophoblast cells and played an important role in the syncytiotrophoblast formation in primates, rodents, lagomorphs, ruminants, and carnivores [6,8,11–13]. Even with no evidence of syncytiotrophoblast formation, the horse placenta contains some invasive cells called the chorionic girdle trophoblasts which, in the early gestation period, invade the uterus of the pregnant mare to form some structures in the superficial endometrium known as endometrial cups [27,28]. These cups are composed of binucleate trophoblast cells that share many features with the syncytiotrophoblast of the human placenta [29]. In the Author’s opinion, a specific sampling of endometrial cups could have been more informative not just for their "anatomic similarity" with the syncytiotrophoblast of human placenta but also because these cups secrete large quantities of equine gonadotrophic hormone (eCG). In human medicine, ERV-3 *env* was found to be especially expressed in hormone-producing organs [30,31]. These findings suggest that it may play a critical role in the process of hormone secretion. For these reasons, it is probable that we could have found higher expression levels if we had sampled the equine endometrial cups.

Moreover, the timing of sampling could have a role on level of ERV *env* expression as shown by Heidmann et al., in rabbits [13] where the placenta-specific expression decreases with gestational age. In our study, we were unable to test placenta at different times of gestation as the fetal parts of the placentae were collected after an eutocic delivery at the end of physiological gestation (near 330 days) whereas samples from pregnant mares killed at several gestation times were not used for ethical reasons. However, in horses, the placental barrier maintains an epitheliochorial arrangement during the course of pregnancy. The most considerable alteration on the maternal side of the placenta is a thinning of the uterine epithelium with a reduction to one-third of its original height by the 300^th^ day of pregnancy [32].

Despite some limitations of the present study, the fetal part of placental expression level of EqERV *env* RNAs suggests a possible role in placental tissue. Moreover, because in horses there is no syncytiotrophoblast layer at their maternal-fetal interface, a possible immunological role of this EqERV in relation with maternal-fetal tolerance could be hypothesized. An interesting feature of syncytins proteins is indeed the presence of an immunosuppressive domain (ISD). These proteins may be involved in suppression of maternal immunoreactivity against the allogeneic feto-placental unit [33]. In fact, some authors agree that this immune tolerance could even be the original function of syncytins prior to their fusogenic capacity [2].

Moreover, in the human field, ERVs are hypothesised to be involved in diseases such as multiple sclerosis [34] and cancer [35] and they have a role in the evolution of the host genome, by, for example, changing nearby gene expression [36]. Although transcriptional interference of ERVs in gene expression is less documented in mammals than in plants, the knowledge in this area was recently improved with the findings that ERVs can influence the expression of mammalian resident genes by disrupting transcriptional termination [36]. Our future studies will be directed toward the understanding of how EqERV could modify the expression of surrounding genes.

In the present study, an overexpression of the endogenous retroviral sequences has been shown in the physiological fetal part of the placentae but a possible role in pathological conditions cannot be excluded. In human medicine, some authors hypothesized that several gestational diseases affecting fetuses, or even choriocarcinoma, could be due to defective syncytiotrophoblast formation because of an altered expression of *syncytin* genes [2,37,38]. A challenge for future research will be studying the alterations of the expression of the ERVs sequences in horse pregnancy disease as well.

In our opinion, an interesting outcome of the present investigation is the expression of a retroviral *env* gene with syncytin-like properties outside the well-characterized *syncytins* that are associated with the invasive placentation types and with the syncytiotrophoblast formation. Further research is required to show and characterize in which specific placental site the expression takes place and to understand the role of the *env* protein in equine placental morphogenesis.

## Supporting Information

S1 Fig Expression of EqERVs *env* RNAs in normal equine tissues. Each value was normalized to the amount of the two reference genes (*β-actin* and *GAPDH*) and was exhibited as percent of maximum.
(XLSX)

S1 Table Full-length candidates EqERVs. (XLSX)

S2 Table Descriptive statistics. (XLSX)
